# Cardiac Biomarker Complete Response in Light Chain Amyloidosis: How Much of the Heart Is Captured?

**DOI:** 10.1016/j.jaccao.2026.02.003

**Published:** 2026-04-21

**Authors:** Alexander H. Gunn, Cristiana Costa Chase, Michel G. Khouri

**Affiliations:** a Division of Cardiology, Department of Medicine, Duke University Health System, Durham, North Carolina, USA; b Division of Hematologic Malignancies and Cellular Therapy, Department of Medicine, Duke University Health System, Durham, North Carolina, USA

**Keywords:** amyloidosis, biomarkers, echocardiography, outcomes, treatment

**Figure:**
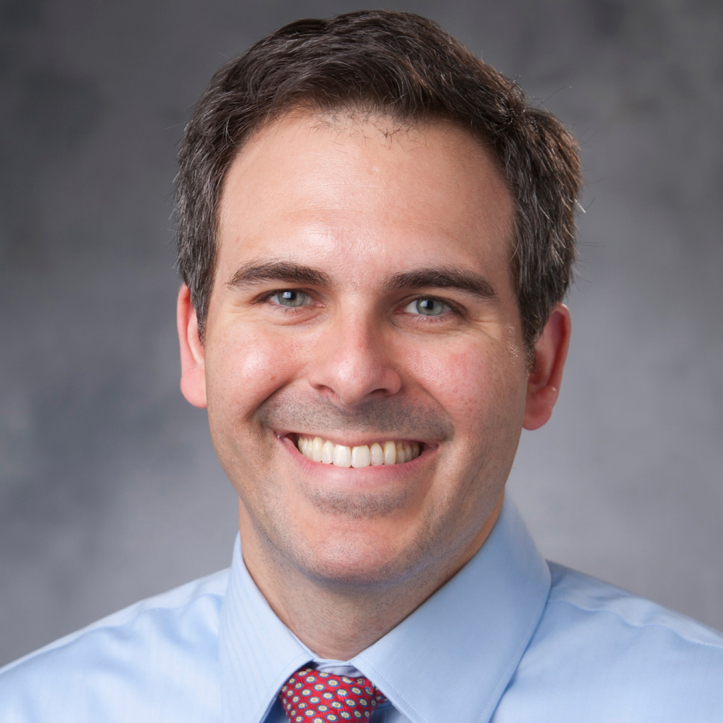

Immunoglobulin light chain (AL) amyloidosis is a plasma cell dyscrasia in which the extent of cardiac involvement is the primary determinant of prognosis. Misfolded ALs aggregate into toxic oligomers and amyloid fibrils that deposit in the myocardium, leading to restrictive cardiomyopathy, heart failure, conduction abnormalities, and arrhythmias.^1^ Cardiac involvement is assessed using validated staging criteria for AL amyloidosis that incorporate blood-based biomarkers for myocardial injury and stress, such as troponin T and N-terminal pro–B-type natriuretic peptide (NT-proBNP).^1^ Advances in plasma cell–directed therapy over the past 2 decades have improved survival rates, making the long-term effects of cardiac disease burden and interdisciplinary care increasingly important.

In this issue of *JACC: CardioOncology*, Muchtar et al^2^ describe a cohort of patients with AL cardiac amyloidosis (CA) from a single center between 2004 and 2023 who achieved cardiac biomarker complete response (CR). Cardiac involvement was determined on the basis of imaging findings consistent with CA on echocardiography or cardiac magnetic resonance imaging, supplemented by tissue biopsy confirming AL amyloid deposits. Patients were included if they had elevated levels of natriuretic peptides, specifically NT-proBNP (>650 pg/mL) or B-type natriuretic peptide (>150 pg/mL), at baseline. Cardiac biomarker CR was defined by the sustained normalization of NT-proBNP to ≤350 pg/mL or B-type natriuretic peptide to ≤80 pg/mL for at least 12 months.

Of 1,342 AL CA patients with elevated natriuretic peptides, 63 patients (4.7%) achieved cardiac biomarker CR during the 20-year study period. Between the first half (2004-2013) and second half (2013-2024) of the study, the proportion of patients achieving cardiac biomarker CR rose from 3.3% to 6.4%; during this time, the use of bortezomib-based and daratumumab-based regimens increased, while the frequency of autologous stem cell transplantation declined. The median time to first documented cardiac biomarker CR was 20.6 months (Q1-Q3: 11.8-27.3 months) from treatment initiation, and 96% achieved CR by 60 months. Within the first year, 97% of patients achieved hematologic very good partial response or CR, underscoring that cardiac biomarker improvement trails light chain suppression. At CR, the median NT-proBNP concentration was 265 pg/mL (Q1-Q3: 220-307 pg/mL), representing an 86% reduction from the pretreatment median of 1,977 pg/mL; troponin T and high-sensitivity troponin T decreased by 75% and 52.6%, respectively. On echocardiography, interventricular septal wall thickness decreased; the proportion of patients with interventricular septal wall thickness ≤12 mm rose from 26.2% to 43.6%. Indexes of myocardial performance increased, including left ventricular longitudinal strain, stroke volume index, and the proportion of patients with left ventricular ejection fraction ≥ 55% and estimated right ventricular systolic pressure < 35 mm Hg. Patients who achieved cardiac biomarker CR were generally younger (median age 57 vs 65 years), had a higher rate of kappa isotype (36.5% vs 24.4%), had better renal function (median estimated glomerular filtration rate 73 vs 59 mL/min/1.73 m^2^), and were more often diagnosed at an earlier cardiac stage (57.1% vs 39.4% at Mayo 2004 stages I and II).

The investigators report outstanding survival in this cohort. The median survival among patients who achieved cardiac biomarker CR was 9.3 years from treatment initiation, and the estimated 5- and 10-year survival rates were 98% and 92.1%, respectively. Impressively, overall survival in the cardiac biomarker CR cohort was similar to that of the general population matched for age, sex, and treatment initiation year. While positioning cardiac biomarker CR as a powerful prognostic marker, these findings also highlight its limitations as a surrogate for long-term cardiac recovery. Muchtar et al^2^ observed that despite robust biomarker improvement, AL CA patients remained susceptible to long-term cardiac risks. Notably, 14% experienced NT-proBNP progression after a median of 7.6 years (Q1-Q3: 3.5-10.2 years) from the initial cardiac biomarker CR, with cases characterized by worsening ejection fraction, new-onset atrial fibrillation, and progressive valvular disease.

Despite prognostic improvements, the ongoing cardiovascular risks from light chain amyloid deposition in the heart cannot be overlooked (Figure 1). The term *cardiac biomarker CR* is derived from earlier work by Muchtar et al^3^ and mirrors hematologic response criteria in AL amyloidosis, in which CR signifies near complete eradication of malignant plasma cells. A key distinction in AL CA is that although current therapies effectively halt further amyloid deposition by suppressing the source plasma cell clone, the majority of AL amyloid fibrils deposited in the heart remain, continuing to disrupt tissue architecture and exerting direct cytotoxic effects.^1^^,^^4^ Biomarker improvements observed in this study may therefore reflect favorable myocardial remodeling and compensatory changes following light chain suppression rather than true reversal of amyloid-related structural heart disease.

**Figure 1:**
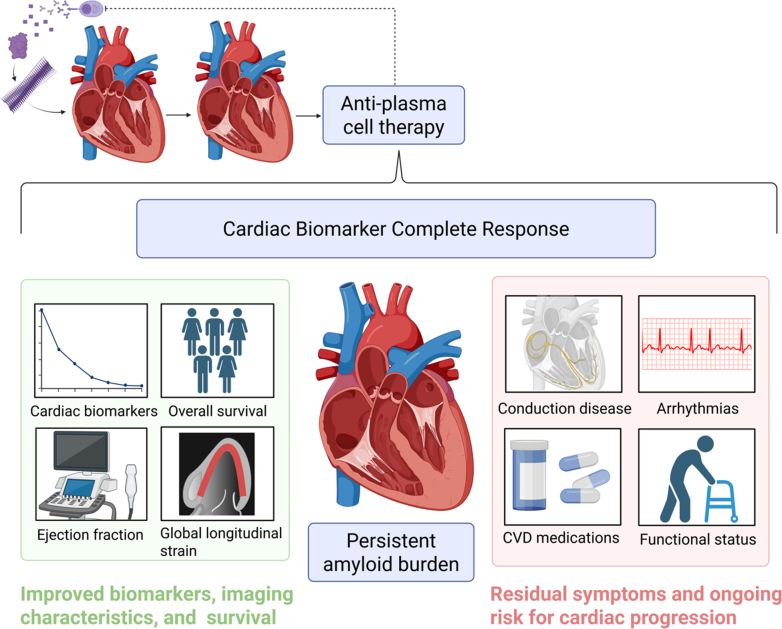
Effects of Cardiac Biomarker Complete Response in Patients With Light Chain Cardiac Amyloidosis Immunoglobulin light chain amyloidosis originates from an abnormal plasma cell population in the bone marrow that produces unstable light chains, which misfold and deposit as amyloid in the heart. Plasma cell–directed therapy can improve cardiac function and survival for patients who achieve cardiac biomarker complete response. However, amyloid deposits remain, and ongoing cardiovascular care is necessary because of persistent risks for heart failure and conduction and rhythm abnormalities with potential impacts on functional capacity and quality of life. Figure created in BioRender (https://BioRender.com/kjrsgj5). CVD = cardiovascular disease.

From a patient perspective, cardiac biomarker CR may not fully encapsulate ongoing symptoms or future cardiovascular risk. A focus on biomarker response rather than patient-centered outcomes, such as functional status, exercise tolerance, and quality of life, risks underestimating the burden of residual AL CA on patients. Notably, one-third of patients continued loop diuretic use at the time of cardiac biomarker CR, at a median daily dose of 0.5 mg/kg furosemide equivalents, suggesting that heart failure symptoms persisted after biomarker levels had normalized. In comparison, patient-centered variables have been effectively incorporated in transthyretin CA staging systems; for instance, the Columbia University score considers daily loop diuretic dose, NYHA functional class, and cardiac biomarkers.^5^ Advancements in multimodal imaging techniques, such as ^18^F-florbetapir positron emission tomography, offer promise for quantifying myocardial amyloid burden.^1^^,^^4^^,^^6^ Future definitions of cardiac response in AL amyloidosis will likely become more precise and personalized by integrating biomarkers, patient-centered measures, and imaging data.

As patients live longer, attention to long-term morbidity and cardiovascular complications of AL CA is increasingly important, even among patients with cardiac biomarker CR. Muchtar et al^2^ highlight the risk for cardiac biomarker progression following CR, complementing previous studies that reported, among patients after diagnosis, that about 12% developed new-onset atrial fibrillation within 1 year, 8.4% required permanent pacemaker implantation within 3 years, and up to 35% had nonsustained ventricular tachycardia on ambulatory monitoring.^7^, ^8^, ^9^ Future studies should comprehensively evaluate cardiac comorbidities over extended periods to guide surveillance and management strategies.

Finally, achieving truly complete cardiovascular response in AL CA will likely require therapies that can reverse amyloid deposition and/or repair its detrimental effects on the heart. Antifibril depleter therapies target amyloid removal through immune-mediated mechanisms, including antibody-dependent phagocytosis and direct enzymatic degradation.^6^ Two monoclonal antibody candidates, birtamimab and anselamimab, have been evaluated in clinical trials, but results were disappointing: the phase 3 trial of birtamimab was halted for futility, and anselamimab failed to meet its primary endpoint.^10^^,^^11^ Although biologic rationale for promoting amyloid clearance remains compelling, clinical translation in AL CA has proved challenging.

In summary, the study by Muchtar et al^2^ reveals that patients who achieve cardiac biomarker CR have survival rates comparable with that of the general population, representing a remarkable therapeutic milestone in AL CA. These results underscore the importance of early detection and effective plasma cell–directed therapy. However, cardiac biomarker CR does not exclude ongoing cardiomyopathy, which requires continued surveillance and management of heart failure symptoms, arrhythmias, and conduction abnormalities that can limit functional capacity and quality of life. With improving survival, future efforts should prioritize addressing long-term cardiovascular complications and developing therapies that facilitate structural recovery from amyloid deposition for a truly complete cardiac response.

## Funding Support and Author Disclosures

Dr Khouri has acted as a researcher for Alnylam Pharmaceuticals, AstraZeneca, Attralus, BridgeBio Pharma, Intellia Therapeutics, Ionis Pharmaceuticals, and Pfizer; and has acted as a consultant, an advisor, or a speaker for Alnylam Pharmaceuticals, AstraZeneca, BridgeBio Pharma, and Pfizer. All other authors have reported that they have no relationships relevant to the contents of this paper to disclose.

## References

[1] Sanchorawala V. (2024). Systemic light chain amyloidosis. N Engl J Med.

[2] Muchtar E., Geyer S., Dispenzieri A. (2026). Cardiac biomarker complete response in AL amyloidosis: characteristics, cardiac recovery, and survival of 63 patients. JACC CardioOncol.

[3] Muchtar E., Dispenzieri A., Wisniowski B. (2023). Graded cardiac response criteria for patients with systemic light chain amyloidosis. J Clin Oncol.

[4] Benz D.C., Clerc O.F., Cuddy S.A.M. (2025). Changes in myocardial light chain amyloid burden after plasma cell therapy. JACC Cardiovasc Imaging.

[5] Cheng R.K., Levy W.C., Vasbinder A. (2020). Diuretic dose and NYHA functional class are independent predictors of mortality in patients with transthyretin cardiac amyloidosis. JACC CardioOncol.

[6] Fontana M., Ioannou A., Cuddy S. (2025). The last decade in cardiac amyloidosis: advances in understanding pathophysiology, diagnosis and quantification, prognosis, treatment strategies, and monitoring response. JACC Cardiovasc Imaging.

[7] Porcari A., Rossi M., Cappelli F. (2022). Incidence and risk factors for pacemaker implantation in light-chain and transthyretin cardiac amyloidosis. Eur J Heart Fail.

[8] Choi Y.J., Kim D., Rhee T.M. (2023). Left atrial reservoir strain as a novel predictor of new-onset atrial fibrillation in light-chain-type cardiac amyloidosis. Eur Heart J Cardiovasc Imaging.

[9] Chen Z., Shi A., Dong H. (2024). Prognostic implications of premature ventricular contractions and non-sustained ventricular tachycardia in light-chain cardiac amyloidosis. Europace.

[10] Gertz M.A., Cohen A.D., Comenzo R.L. (2023). Birtamimab plus standard of care in light-chain amyloidosis: the phase 3 randomized placebo-controlled VITAL trial. Blood.

[11] Bowden M. Update on CARES Phase III clinical programme of anselamimab in light chain amyloidosis. https://www.astrazeneca.com/media-centre/press-releases/2025/update-on-anselamimab-in-al-amyloidosis.html.

